# Limitation of tube thoracostomy in treating pneumothorax in COVID-19 infected patients. A retrospective cohort study

**DOI:** 10.1016/j.amsu.2022.104171

**Published:** 2022-07-18

**Authors:** Amer Al-Ani, Heba AbuZayda, Hala Ahmed, Majd Alobied, Nijamudeen Kabeer, Anmar Atasi, Vidya Jakapure, Toufic Dabit, Mohammad Al-Ani

**Affiliations:** a College of Medicine, Ajman University, Ajman, United Arab Emirates; b College of Medicine, Sharjah University, Sharjah, United Arab Emirates; c Kings College Hospital, London, United Kingdom; d Sheikh Khalifa Medical City Ajman, Ajman, United Arab Emirates; e School of Medicine, University of Jordan, Jordan

**Keywords:** COVID-19, Pneumothorax, Plural effusion, Barotrauma, Tube thoracostomy, Trauma

## Abstract

**Background:**

COVID^19^ infection is caused by the highly contagious SARS-CoV-2(Severe acute respiratory syndrome coronavirus 2). The first outbreak of this infection was in Wuhan, China in December 2019. Since then, it has spread rapidly across the world, with more than 100000 new cases each day. Among those infected with SARS-COV-2 up to 20% develop severe disease requiring hospitalization. Among those who are hospitalized, one quarter will need ICU admission. Admission to the ICU is due to respiratory failure or pneumonia. The pneumonia associated with COVID^19^ infection may lead to respiratory failure requiring endotracheal intubation and mechanical ventilation. An important complication of mechanical ventilation is barotrauma. Barotrauma appears to be common in COVID^19^ patients. Pneumothorax developed in 25% of COVID^19^ patients who had barotrauma. In COVID^19^ the percentage of patients with mild symptoms who develop a pleural effusion is 8% compared to 28% in patients who are critically ill. Most of the COVID^19^ infected that have a pneumothorax or pleural effusion need a thoracostomy. In trauma cases most, thoracic injuries (leading to pneumothorax or hemothorax) are effectively treated with tube thoracostomy.

**Objectives:**

First objective is to compare the therapeutic effect of tube thoracostomy on COVID^19^ infected patients who have pneumothorax or pleural effusion to those non-COVID^19^ infected patients who had traumatic pneumothorax or pleural effusion treated by tube thoracostomy. Second objective is to study the morbidity associated with tube thoracostomy in COVID^19^ infected patients who have pneumothorax or pleural effusion.

**Patients and methods:**

This study was conducted in Sheikh Khalifa medical city Ajman, United Arab Emirates. It is a descriptive, observational, retrospective cohort study. One hundred patients were recruited from the January 1, 2020 to the December 31, 2020. Patients were divided into two groups. First group includes fifty adult COVID ^19^ infected patients who had no trauma. Second group includes fifty adult COVID^19^ infection free patients who had trauma. **Inclusion criteria for the first group**: COVID ^19^ infected patients with an age equal to or above 18 years, of both genders, with history of pneumothorax, pleural effusion or both of them, needed insertion of thoracostomy chest tube. **Inclusion criteria for the second group:** Patients with an age equal to or above 18 years, of both genders, with history of traumatic pneumothorax, pleural effusion (hemothorax) or both of them, needed insertion of thoracostomy chest tube. **Exclusion criteria for the first group**: Children, Adult COVID^19^ infected patients who didn't have pneumothorax or plural effusion, adult COVID^19^ infected patients who had pneumothorax or plural effusion without a need for tube thoracostomy. **Exclusion criteria for the second group**: Adult non-COVID^19^ infected patients who had trauma, but didn't have pneumothorax or pleural effusion, adult non-COVID^19^ infected patients who had traumatic pneumothorax or pleural effusion without a need for tube thoracostomy. The collected data was revised, coded, tabulated and introduced to a PC using Statistical package for Social Science (SPSS 25). Mann Whitney Test (*U* test) was used to assess the statistical significance of the difference of a non-parametric variable between two study groups. Chi-Square test was used to examine the relationship between two qualitative variables. Fisher's exact test was used to examine the relationship between two qualitative variables when the expected count is less than 5 in more than 20% of cells.

**Results:**

Most of patients in trauma group (group 2) were with the age range of 20–40-year (58.8% of patients) P value was significant (<0.001). In COVID ^19^ infected patients' group (group 1) the age range was 40–60 year (50%of patients). P Value (<0.001) was significant too. Male was the dominant gender in group 2 (96.1% of patients were male), while in group1 (78% of patients were male), P Value was significant (0.007). No co-morbidities (diabetes, hypertension, ischemic heart disease, Asthma and dyslipidemia) were detected in group 2 (0.0%). Co-morbidity were detected in 76% of patients in group 1, P Value was significant (<0.001). Hemothorax occurred in 37.3% of patients in group 2, and no cases of hemothorax was detected in group 1. P Value was significant (<0.001). Complications of chest tube insertion took place in group 2 as follows; tube malposition in 13.7% of patients, tube blockade in 3.9% of patients. The percentage in group 1 was as follows tube malposition in 16% of patients, tube blockade in 18%. The difference between the two was not significant for tube malposition (P value 0.748) and significant for tube blockade (P value 0.023). Subcutaneous emphysema occurred in 15.7% of patients in group 2 and in 15.7% of patients in group 1. The difference was not significant (P value was 0.118). Acquired bronchopleural fistula occurred 2.0% of group 1 cases. No cases of this fistula were documented in group 2. Number of chest tubes needed to be inserted in group 2 patients was as follows (one chest tube in: 74.5% of patients, two chest tubes in: 23.5% of patients. Three chest tubes or more in 2% of patients). While in group1 patients’ number of chest tubes needed to be inserted was (one in 56% of patients, two in 30% of patients. Three or more in 14% of patients). The difference was significant only in those who required insertion of three chest tubes or more (P value was 0.028). The median duration needed to keep a chest tube was 3 days in group 2, and 7 days in group 1. The difference between the two was significant (P value was 0.000). Death was the fate of 3.9% of patients in group 2 and in 64% of patients in group 1. The difference was significant (P value was< 0.001)

**Conclusion:**

Therapeutic effect of tube thoracostomy in treating Adult COVID^19^ patients who had pneumothorax or pleural effusion is less than that used in treating trauma non-COVID^19^ patients who had pneumothorax or plural effusion. Morbidity and mortality related to tube thoracostomy applied to treat pneumothorax or pleural effusion in adult COVID^19^ patients is more than that in trauma non COVID ^19^ patients.

## Highlights

• Barotrauma appears to be common in COVID^19^ patients on of mechanical ventilation.
• Pneumothorax developed in 25% of COVID^19^ patients who had barotrauma.
• Most of COVID19 pneumothorax need tube thoracostomy.
• Therapeutic effect of thoracostomy in managing pneumothorax in COVID^19^ is not as good as that used in managing pneumothorax in trauma patients.

## 1 Introduction

The number of people infected with COVID^19^ virus worldwide is increasing on daily bases. It reaches more than 261 million cases [1]. Among them; up to 20% develop severe disease requiring hospitalization [2,3] Although rates vary, among those who are hospitalized, up to one quarter need intensive care unit admission, representing approximately 5–8% of the total infected population. Pneumonia associated with novel coronavirus disease 2019 (COVID^19^) may lead to respiratory failure with profound hypoxemia requiring endotracheal intubation and mechanical ventilation [4]. An important complication of mechanical ventilation is barotrauma. Barotrauma appears to be high in COVID^19^ patients. Pneumothorax developed in up to 25% of COVID^19^ who had barotrauma However it develops only in 2% of patients with other causes of ARDS [5,6]. Percentage of pleural effusion in COVID^19^ patients with mild symptoms is only 8% compared to 28% in patients critically ill with COVID^19^ infection [7]. Insertion of a chest drain is widely recommended as the gold standard and mainstay of treatment in traumatic pneumothorax [8,9]. For critically ill patients on positive pressure ventilation, although controversial, it is currently recommended to place a tube thoracostomy when a pneumothorax is observed [10]. Due to the limited knowledge of lung histopathology with COVID^19^, it is unknown how well the diseased lung tissue will spontaneously heal and re-expand without intervention [10].

This study was conducted to compare the therapeutic effect of tube thoracostomy on COVID^19^ infected patients who have pneumothorax or plural effusion to those non- COVID^19^infected patients who had traumatic pneumothorax or plural effusion treated by tube thoracostomy and to Study morbidity of tube thoracostomy on COVID^19^infected patients who have pneumothorax or pleural effusion.

## 2 Patients and Methods

**This work has been reported in line with the STROCSS criteria** [11]^**.**^ This study registration unique identifying number (UIN) is researchregistry7938.

This study was conducted in Sheikh Khalifa medical city Ajman, United Arab Emirates. It is a descriptive, observational, retrospective cohort study. One hundred patients were recruited from the January 1, 2020 to the December 31, 2020. Patients were divided into two groups. First group includes fifty adult COVID ^19^ infected patients who had no trauma. Second group includes fifty adult COVID^19^ infection free patients who had trauma. **Inclusion criteria for the first group:** COVID ^19^ infected patients with an age equal to or above 18 years, of both genders, with history of pneumothorax, pleural effusion or both of them, needed insertion of thoracostomy chest tube. **Inclusion criteria for the second group:** Patients with an age equal to or above 18 years, of both genders, with history of traumatic pneumothorax, pleural effusion (hemothorax) or both of them, needed insertion of thoracostomy chest tube. **Exclusion criteria for the first group**: Children, Adult COVID^19^ infected patients who didn't have pneumothorax or plural effusion, adult COVID^19^ infected patients who had pneumothorax or plural effusion without a need for tube thoracostomy. **Exclusion criteria for the second group**: Adult non-COVID^19^ infected patients who had trauma, but didn't have pneumothorax or pleural effusion, adult non-COVID^19^ infected patients who had traumatic pneumothorax or pleural effusion without a need for tube thoracostomy. The collected data was revised, coded, tabulated and introduced to a PC using Statistical package for Social Science (SPSS 25). Mann Whitney Test (*U* test) was used to assess the statistical significance of the difference of a non-parametric variable between two study groups. Chi-Square test was used to examine the relationship between two qualitative variables. Fisher's exact test was used to examine the relationship between two qualitative variables when the expected count is less than 5 in more than 20% of cells.

## 3 Results

**Fig. 1:**
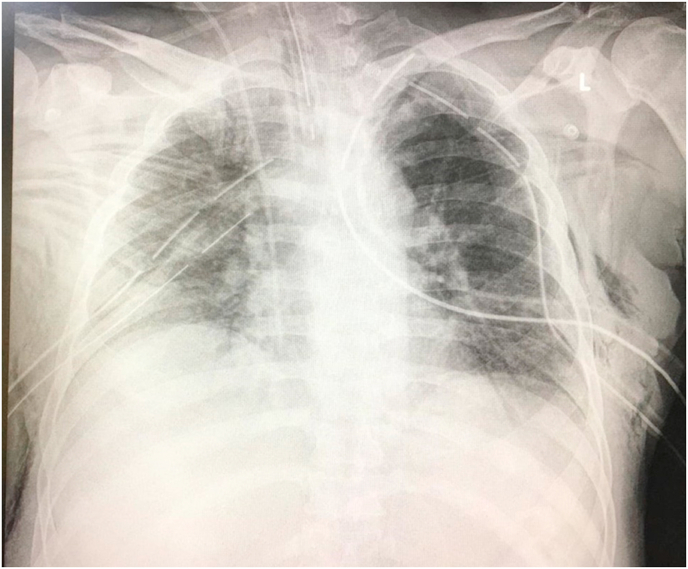
Four chest tubes were inserted to manage pneumothorax in COVID [19] infected patient following barotrauma.

**Fig. 2:**
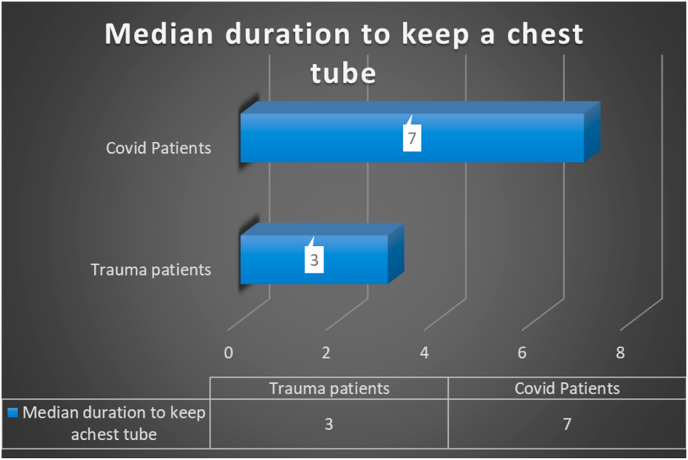
Comparison between the median duration (in days) required for a chest tube to manage pneumothorax in trauma patients' group and the median duration required in COVID [19] infected patients' group.

Most of patients in trauma group (group 2) were with the age range of 20–40-year (58.8% of patients) P value was significant (<0.001). In COVID [19] infected patients' group (group 1) the age range was 40–60 year (50%of patients). P Value (<0.001) was significant too. Male was the dominant gender in group 2 (96.1% of patients were male), While in group1 (78% of patients were male), P Value was significant (0.007). No co-morbidities (diabetes, hypertension, ischemic heart disease, Asthma and dyslipidemia) were detected in group 2 (0.0%). Co-morbidity were detected in 76% of patients in group 1, P Value was significant (<0.001). Hemothorax occurred in 37.3% of patients in group 2, and no cases of hemothorax was detected in group 1. P Value was significant (<0.001). Complications of chest tube insertion took place in group 2 as follows; tube malposition in 13.7% of patients, tube blockade in 3.9% of patients. The percentage in group 1 was as follows tube malposition in 16% of patients, tube blockade in 18%. The difference between the two was not significant for tube malposition (P value 0.748) and significant for tube blockade (P value 0.023). Subcutaneous emphysema occurred in 15.7% of patients in group 2 and in 15.7% of patients in group 1. The difference was not significant (P value was 0.118). Acquired bronchopleural fistula occurred 2.0% of group 1 cases. No cases of this fistula were documented in group 2. Number of chest tubes needed to be inserted in group 2 patients was as follows (one chest tube in: 74.5% of patients, two chest tubes in: 23.5% of patients. Three chest tubes or more in 2% of patients). While in group1 patients’ number of chest tubes needed to be inserted was (one in 56% of patients, two in 30% of patients. Three or more in 14% of patients). The difference was significant only in those who required insertion of three chest tubes or more Fig. 1 (P value was 0.028). The median duration needed to keep a chest tube was 3 days in group 2, and 7 days in group 1 Fig. 2. The difference between the two was significant (P value was 0.000). Death was the fate of 3.9% of patients in group 2 and in 64% of patients in group 1. The difference was significant (P value was< 0.001).

## 4 Discussion

**Fig. 3:**
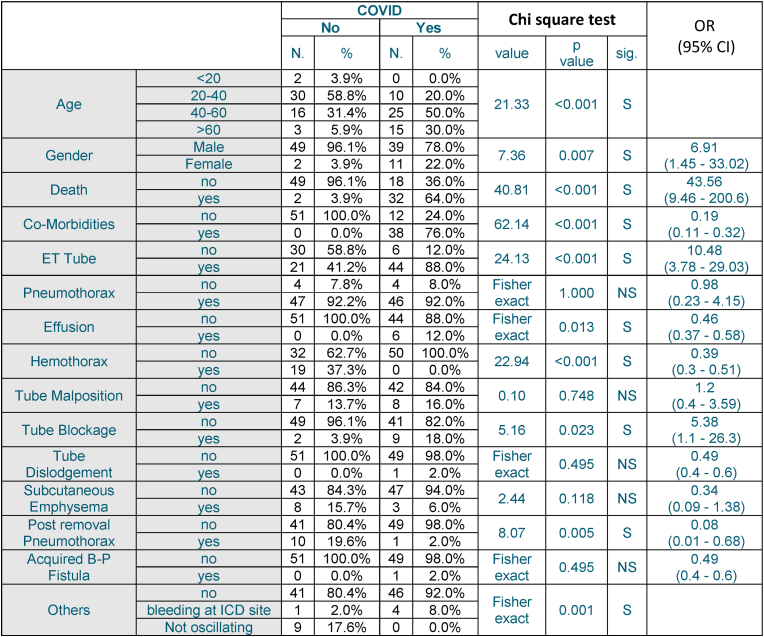
Summary of the findings in COVID and trauma groups in of this study.

All the statistical findings of this study were summarized in Fig. 3. Pneumothorax is a common finding after trauma. In general, pneumothorax which is visible on plain chest X-ray following trauma should be treated with the placement of an intercostal drain [11,12]. In our institute, each patient with COVID^19^ disease who is in need for admission to ICU will have a chest radiograph on an almost daily basis to help guide clinical decisions and medical management. A CT scan of the chest will be done as well if patient started to have unexpected deterioration despite a proper management of his/her COVID^19^ infection and its complications. Pneumothorax can be classified into spontaneous and traumatic (iatrogenic is one type of traumatic pneumothorax). Chest tube thoracostomy is used as a primary management for cases of traumatic pneumothorax. Iatrogenic pneumothorax is mostly related to mechanical ventilation. Barotrauma caused by mechanical ventilation is more common in patients with an underlying lung disease, such as chronic obstructive pulmonary disease or ARDS [12,13]. In COVID^19^ patients with severe involvement of the lung parenchyma, pulmonary compliance is reduced due to pathological changes such as edema, vascular congestion, and inflammation [13,14] As a result, it is possible that over-inflation and high PEEP (Positive end expiratory pressure) in such hypoplastic and fibrotic lungs can lead to alveolar rupture and barotrauma [14,15]. The reported prevalence of barotrauma in COVID^19^ patients is 15% [15,16]. In barotrauma-related pneumothorax occur in mechanically ventilated patients with COVID^19^ infection, tube thoracostomy and drainage has shown satisfactory outcomes [14,15]. In this study; this finding was not the same; more than one tube thoracostomy were needed to be applied in most cases of COVID^19^ infection who developed pneumothorax due to barotrauma. In relation to age and gender, this study showed that most of trauma cases who developed pneumothorax were young (58.8% of cases) with an age range of 20–40 year and most of them were male (96.1%). However, the age range of COVID^19^ patient who developed pneumothorax in this study was 40–60 year in 50% and was >60 year in 30% of cases, further to that most of the cases were male (78%). This is similar to other studies [16,17,17,18]. This is similar to the findings in Housman B et al. study [18,19]. Comorbidities like hypertension, diabetes, ischemic heart disease, asthma, hyperlipidemia and Arrhythmia were absent in all cases of trauma (51 patient) in this study; but it was present in 76% of the patients (38 out of 50 patients) in COVID^19^ infection group. This can explain the high mortality rate in COVID [19] patients’ group in this study. In this study 37.7% of trauma group patients (group2) had hemothorax in addition to pneumothorax. Pneumothorax after trauma is commonly associated with bleeding [11,12]. On the contrary; no case of hemothorax was documented in COVID^19^ cases. Desnos et al. documented 4 cases of COVID [19] spontaneous hemothorax [19,20]. No cases of effusion were documented in trauma cases in this study. But it was documented in 12% of COVID cases (6 patients). This is similar to a recent study which found that pleural effusion took place in 10.3% of COVID^19^ patients and that those refractory patients had a higher incidence of pleural effusion than general COVID^19^ patients, suggesting a more obviously inflammatory response in the lung [20,21]. Chest tube insertion complications in this study were as follows. Tube malposition noticed in 7 patients (13.7%) when applied on trauma patients and 8 patients (16%) when applied in COVID^19^ patients. Manuel F et al. documented chest tube malposition in 42% of patients who had emergency admission after trauma and needed chest tubes. In his study Manuel found that chest tubes were in the pleural space in 58% of the cases; while malposition were intrafissural positions in (27%), intraparenchymal positions in (11%) and extra pleural positions in (4%) [21,22]. In his study Kun-Eng Lim et al. documented 28 chest tube malposition among the 76 chest tubes that were placed in 54 patients. Among the 28 malposition, 23 tubes were in the intrathoracic location (20 intraparenchymal; 3 intrafissural) and 5 tubes were in the extra thoracic location (4 in mediastinum; 1 in chest wall) [22,23]. Tube clotting, especially in smaller tubes, is a common cause of non-functioning drainage [23,24]. In this study; sizes of chest tubes used to deal with pneumothorax, effusion or haemothorax in trauma and in COVID^19^ cases were ranging between 24F–36F; actually, sometimes the larger size tubes were preferred in cases of pneumothorax with big surgical emphysema or if there is repeated obstruction of tubes. Chest tube blockage was developed more in COVID^19^ patients, it happens in in 9 patients (18%); while it was documented in only 2 of trauma patients (3.9%). Chest tube dislodgement after insertion is a common occurrence. Chest tube movement is inevitable, especially in critically ill patients who are frequently repositioned for procedures [24,25] AA Talpur et al. reported a ratio of 1% of dislodged chest tubes in patients having any pathology related to chest cavity and underwent chest intubation [25,26] In this study the ratio was almost similar (2% in COVID^19^ patients, o% in trauma patients). Subcutaneous emphysema in this study had happened after chest tube insertion as follows (15.7% in trauma patient and in 3 (6%) in COVID^19^ patients. AA Talpur et al. reported a ratio of 3% [25,26]. TW Khanzada et al. found subcutaneous emphysema in 5/105 patients, which resolved spontaneously within few days [26,27]. Number of chest tube inserted for cases of trauma in this study was as the following: 1 tube in 38 patients (74.5%) 2tubes in 12 (23.5%) 3 and more in 1patient (2%). This depends on the severity and the extension of the trauma they had. On the other hand, in cases of COVID^19^ the number of chest tube inserted was as the following: 1 tube in 28 patients (56%) 2tubes in 15 (30%) 3 and more in 7 patients (14%). The difference between the two was statistically significant (P value 0.018) the increased number of tubes needed in COVID ^19^ patients is due to the wide broncho-plural fistula that they usually developed because of barotrauma. Zhang et al. in a meta-analysis study on the single chest tube versus double chest tube application after pulmonary lobectomy reached a conclusion that: Compared with the double chest tube, the single chest tube significantly decreases amount of drainage, duration of chest tube drainage, pain score, the number of patients who need thoracentesis, and cost. Although there is convincing evidence to confirm the results mentioned herein, they still need to be confirmed by large-sample, multicenter, randomized, controlled trials [27,28]. Tawil I et al. showed that doctors would remove a chest tube a minimum of 5–7 days after its insertion in patients who were under mechanical respiration and had met the conditions for chest tube removal [28,29]. The median duration to keep a chest tube in trauma patient in this study was 3 and the Interquartile range was 2–5. In COVID^19^ complicated cases median for chest tube stay was 7 and the Interquartile range was 4–15. This finding in COVID ^19^ patients is due to the longer duration of mechanical ventilation needed for these patients. The difference between group 1 and 2 in this study was statistically significant and the P value was 0.000. Death occurred in 2 trauma patients (3.9%), and in 32 COVID^19^ complication patients (64%) with a P value of <0.001, which is statistically significant. All deaths in this study were related to complications of trauma and complications of COVID^19^ infection; they are not related to pneumothorax. This is similar to the findings of Zantah et al. who found that in all cases, mortality (66.6%) was not directly related to the pneumothorax. ^29 30^ Unfortunately, the increased risk of mortality for patients undergoing thoracic surgery with a concomitant active COVID^19^ infection makes the timing of a definitive operation a challenging decision [30,31].This is why all of COVID^19^ infections in this study were not treated surgically. The use of EBVs (endobronchial valve system for BPF (bronchopleural fistula (BPF) with persistent air leaks in SARS-CoV-2 patients who are poor surgical candidates is effective and safe [31,32]. Unfortunately, this mode of treatment was not available in our facility, this is why it was not applied on patients in this study.

## 5 Conclusion

This study concludes that the therapeutic effect of tube thoracostomy in treating adult COVID^19^ patients who had pneumothorax or plural effusion is not as satisfactory as what is mentioned in other studies. Moreover, this study showed that tube thoracostomy in COVID ^19^ patients is less effective than when it is used in treating trauma non- COVID^19^ patients who had pneumothorax or plural effusion. Morbidity and mortality related to tube thoracostomy used to treat pneumothorax or pleural effusion in adult COVID^19^ patients were more than that used to treat pneumothorax or pleural effusion in trauma patients.

## Limitation of this work

It would add to the study if a definitive treatment like VAT (Video-assisted thoracoscopic surgery or EBVs (endobronchial valve system) were possible to be applied on COVID^19^ patient who have broncho-plural fistula. This would reveal whether the limitation in the management of barotrauma in COVID^19^ patient is restricted only to tube thoracostomy, or it's also present when definitive treatment is applied. A comparison study between tube thoracostomy and VAT or EBVs in managing barotrauma in COVID^19^ patients is needed.

## Provenance and peer review

Not commissioned, externally peer-reviewed.

## Funding

This study had granted an internal seed grant form Sheikh Khalifa Medical City Ajman. Ajman. UAE.

## Sources of funding

This research was supported by a seed grant from sheikh Khalifa medical city Ajman. United Arab Emirates.

## Ethical approval

United Arab Emirates. Ministry of Health and Prevention. Research Ethics Committee.

Approval Reference No: MOHAP/DXB-REC/MMM/No. 26/2021.

## Consent

Written informed consent was obtained from the patients for publication of this study and accompanying images. A copy of the written consents is available for review by the Editor-in-Chief of this journal on request”.

## Author statement

Study concept or design: Amer Al-Ani. Data collection: Heba AbuZayda, Hala Ahmed, Majd Alobied, Nijamudeen Kabeer, Anmar Atasi, Amer Al-Ani. Data analysis or interpretation: Amer Al-Ani, Vidya Jakapure. Writing the paper: Amer Al-Ani, Mohammad Amer Al Ani, Toufic Dabit.

## Registration of research studies

1. Name of the registry: research registry
2. Unique Identifying number or registration ID: researchregistry7938
3. Hyperlink to your specific registration (must be publicly accessible and will be checked):

## Guarantor

The Guarantor is the one or more people who accept full responsibility for the work and/or the conduct of the study, had access to the data, and controlled the decision to publish.

## Declaration of competing interest

Authors declare that they have no conflicts of interest to disclose.
